# Identification of a cuproptosis and copper metabolism gene–related lncRNAs prognostic signature associated with clinical and immunological characteristics of hepatocellular carcinoma

**DOI:** 10.3389/fonc.2023.1153353

**Published:** 2023-03-28

**Authors:** Wei Yuan, Jun-hao Xiao, Jian-song Zhang, Ben-liang Mao, Peng-zhen Wang, Bai-lin Wang

**Affiliations:** ^1^ Department of Hepatobiliary Surgery, Guangzhou Red Cross Hospital of Jinan University, Guangzhou, Guangdong, China; ^2^ Department of Clinical medicine, Guizhou Medical University, Guiyang, Guizhou, China; ^3^ Guangzhou Institute of Traumatic Surgery, Guangzhou Red Cross Hospital of Jinan University, Guangzhou, Guangdong, China

**Keywords:** AL590705.3, LINC02870, KDM4A-AS1, MKLN1-AS, hepatocellular carcinoma, immunological characteristics, prognostic signature

## Abstract

**Background:**

The relationship between cuproptosis and HCC is still in the exploratory stage. Long noncoding RNAs (lncRNAs) have recently been linked to the progression of hepatocellular carcinoma (HCC). However, the clinical significance of lncRNAs associated with cuproptosis remains unclear.

**Methods:**

Based on The Cancer Genome Atlas (TCGA) liver hepatocellular carcinoma (LIHC) dataset, we identified characteristic prognostic lncRNAs by univariate, LASSO, and multifactorial regression analysis, and constructed a prognostic signature of cuproptosis-related lncRNAs in HCC. The role of lncRNAs were identified through CCK-8, clone formation in Huh-7 cells with high expression of FDX1. Prognostic potential of the characteristic lncRNAs was evaluated in each of the two cohorts created by randomly dividing the TCGA cohort into a training cohort and a test cohort in a 1:1 ratio. Immune profiles in defined subgroups of cuproptosis-related lncRNA features as well as drug sensitivity were analyzed.

**Results:**

We constructed a multigene signature based on four characteristic prognostic lncRNAs (AL590705.3, LINC02870, KDM4A-AS1, MKLN1-AS). These four lncRNAs participated in the development of cuproptosis. HCC patients were classified into high-risk and low-risk groups based on the median value of the risk score. The receiver operating characteristic curve area under the curve values for 1-, 3-, and 5-year survival were 0.773, 0.728, and 0.647, respectively, for the training cohort, and 0.764, 0.671, and 0.662, respectively, for the test cohort. Univariate and multifactorial regression analyses indicated that this prognostic feature was an independent prognostic factor for HCC. Principal component analysis plots clearly distinguished between low- and high-risk patients in terms of their probability of survival. Furthermore, gene set enrichment analysis showed that a variety of processes associated with tumor proliferation and progression were enriched in the high-risk group compared with the low-risk group. Moreover, there were significant differences in the expression of immune cell subpopulations, immune checkpoint genes, and potential drug screening, which provided distinct therapeutic recommendations for individuals with various risks.

**Conclusions:**

We constructed a novel cuproptosis-associated lncRNA signature with a significant predictive value for the prognosis of patients with HCC. Cuproptosis-associated lncRNAs are associated with the tumor immune microenvironment of HCC and even the efficacy of tumor immunotherapy.

## Introduction

The most common liver cancer, hepatocellular carcinoma (HCC), is one of the leading causes of cancer-related deaths worldwide, and its incidence has been rising in recent years (1–3). Hepatitis B virus, hepatitis C virus, and alcohol consumption are the main causes of HCC (4). Currently, surgery and radiofrequency ablation are the main treatment options for early-stage HCC, while radiotherapy, chemotherapy, and hepatic artery chemoembolization with molecular targeted therapy are the main treatment options for intermediate and advanced HCC (5, 6). However, due to the insidious onset of HCC, the majority of patients receive diagnosis at an intermediate to advanced stage and face significant treatment challenges. Immunotherapy, an emerging treatment modality that selectively kills tumor cells *via* the immune system, has emerged as a key player in the treatment of HCC in recent years. Nivolumab, pembrolizumab, and camrelizumab, for example, improve patients’ overall survival (7–9). Nonetheless, HCC recurrence and metastasis rates remain high, and the prognosis remains poor. According to preliminary WHO estimates, more than one million patients worldwide will die from HCC by 2030 (10). Therefore, it is crucial for clinical decision-making to create a prognostic assessment model for HCC with sensitive predictive efficiency.

Copper, which exists in living things in the forms of Cu+ and Cu2+ and controls a number of crucial physiological and pathological processes in the organism, plays a significant role in the internal environment (11, 12). Numerous studies have revealed a strong link between an imbalance in copper and a number of diseases. For instance, exposure to copper oxide nanoparticles increases the expression of the apoptosis-inducing genes p53 and caspase-3, decreases the potential of the mitochondrial membrane, and induces apoptosis in human HCC HEPG2 cells (13). Moreover, a prospective cohort study has revealed a significant link between atherosclerotic cardiovascular disease and circulating serum copper (14). According to a different study, copper chelated with disulfiram controls the Wnt/β-catenin signaling pathway and stress response to reduce the growth of gastric cancer cells (15). Recent research has shown that “cuproptosis,” a novel mode of cell death distinct from necroptosis, autophagy, pyroptosis, and ferroptosis, is caused by the copper-mediated esterification of proteins in the tricarboxylic acid cycle. It has also been shown that FDX1, which encodes a protein that converts Cu2+ into the more toxic Cu+, plays an important role in the process of copper-induced cell death (16). The connection between copper death and HCC is still under investigation, despite the fact that copper-related diseases have been studied in the past.

Long noncoding RNAs (lncRNAs) are nucleotide transcripts with more than 200 nucleotides in length and account for the majority of RNAs (17, 18). Previously, lncRNAs were regarded as a by-product of transcription. There is growing evidence that lncRNAs are strongly linked to biological processes such as cell division, differentiation, migration, apoptosis, and metabolism (19). In the biological processes of tumorigenesis and growth, lncRNAs are crucial players. For example, the miR-199a-5p/c-Myc axis promotes proliferation and aerobic glycolysis in non-small cell lung cancer (NSCLC) through C-MYC-targeted activation of LINC01123 expression (20). TLNC1 and TRP regulate each other and mediate P53 migration from the nucleus to the cytoplasm, while p53 target gene transcription is inhibited, promoting HCC growth and metastasis (21). Additionally, the interaction between lncRNA-PDPK2P and PDK1 promotes the development of HCC through the PDK1/AKT/caspase 3 signaling pathway and makes it a promising molecular target for HCC treatment (22). P53 upregulates TRINGS when there is a glucose shortage and binds to STRAP to protect tumor cells by blocking the STRAP–GSK3–NF-B pathway (23). Prior to this study, Zhang et al. successfully developed a prognostic model for copper death–related lncRNAs in HCC. Despite its good predictive power, that model did not examine the connection between risk scores and the immune microenvironment of HCC (24). Thus, more research is necessary to clarify the connection between copper death and associated lncRNAs and the immune profile of HCC.

In this study, we compared normal and tumor samples from the TCGA_LIHC set in The Tumor Genome Atlas (TCGA) database. We investigated the differentially expressed copper death–related lncRNAs in HCC, developed a new copper death–related differentially expressed lncRNAs signature (CRDELSig), and assessed the relationship between this signature and clinicopathological features of HCC patients, as well as its prognostic significance. In addition, a nomogram was created to efficiently increase individual prediction of the prognosis of HCC patients. We concluded by investigating immune cell infiltration, immune checkpoint distribution of CRDELSig, and individualized immunotherapeutic agent prediction based on the risk score. Finally, we more closely examined the connection between copper death and associated lncRNAs and the immune microenvironment of HCC, which may help with risk stratification and treatment recommendations for HCC patients.

## Materials and methods

### HCC patients’ data collection and lncRNA acquisition

RNA-sequencing (fragments per kilobase exon model per million mapped, FPKM) data and corresponding clinical characteristics (377 cases in total) of HCC patient samples (including 374 liver tumor tissues and 50 adjacent tissues) were downloaded from TCGA (https://portal.gdc.cancer.gov/). Thirty-two genes associated with cuproptosis and copper ion regulation (collectively referred to as cuproptosis-related genes) were obtained from the Molecular Signature Database (MSigDB) and a previously published paper (16). Gene Ontology(GO)functional enrichment and Kyoto Encyclopedia of Genes and Genomes (KEGG)pathway enrichment analysis were used to identify the biological functions of these 32 genes. Annotation was based on lncRNAs downloaded from Genecode (https://www.gencodegenes.org), and we extracted the expression matrix of 16773 lncRNAs in the TCGA dataset using Strawberry Perl.

### Identification of cuproptosis-related differentially expressed lncRNAs

Pearson correlation analysis was performed to determine the correlations between the expression levels of cuproptosis-related genes and those of lncRNAs to identify the cuproptosis-related lncRNAs. The criteria were |Pearson correlation coefficient| > 0.5 and P < 0.001. The “limma” package in R (version 4.1.1) was used for differential analysis of cuproptosis-related lncRNAs (log |FC| > 2 and P < 0.05), and finally 186 differentially expressed lncRNAs (CRDEL) were obtained for subsequent analysis.

### Clinical data collation and random data grouping

After excluding patients with a survival time below 30 days and patients with missing survival time and survival status, 343 samples with complete clinical survival information were finally retained. Subsequently, 343 clinical samples were randomly divided into a training set (n = 207) and a test set (n = 136) according to the ratio of 6:4, using the “caret” package in R.

### Construction of CRDEL signatures and nomograms, and verification in TCGA queues

We performed a univariate Cox regression analysis (P < 0.05) to identify survival-related lncRNAs, and constructed a cuproptosis-related differentially expressed lncRNAs signature (CRDELSig) for HCC using the least absolute shrinkage and selection operator (LASSO) regression model and multivariate Cox regression analyses in the training cohort. We then verified CRDELSig in the testing cohort and the entire cohort. The risk score for each HCC patient was calculated using the following formula: Risk score = Σ coefficient of (lncRNAi) × expression of (nrlncRNAi).

Using the median risk score in the training cohort as a cutoff value, the HCC patients were divided into two groups, including the high-risk group and the low-risk group. Then, risk scores were calculated for all HCC patients in the test cohort and the entire cohort, who were then divided into high- and low-risk groups based on the same cutoff. Kaplan–Meier survival analyses were performed for the high- and low-risk groups in all three cohorts using the “survival” package. P < 0.05 was considered to indicate statistical significance.

Time-dependent receiver operating characteristic (ROC) curves were constructed to evaluate the predictive accuracy and specificity of the risk model and of different clinicopathological characteristics (age, gender, grade, and stage) using the “timeROC” package.

Furthermore, a nomogram based on the risk score and clinical characteristics (gender, grade, age, stage) was established using the “rms” and “regplot” packages, for the prediction of 1-, 3-, and 5-year overall survival of HCC patients. Then, ROC and calibration analyses were performed to verify the prediction accuracy of the nomogram.

### Cell culture and treatment

HepG2, Hep3B, Huh7, 97L, and 97H were purchased from CellCook (Guangzhou, China). HepG2, Hep3B cells were cultured in MEM supplemented with 10% fetal bovine serum and 1×nonessential amino acid. Huh7, 97L, and 97H cells were cultured in DMEM added 10% fetal bovine serum. All cells were amplified in a 5% CO2 incubatorat 37°C.

For cells treatment, indicated concentration of elesclomol was used to treat Huh-7 cells for 24 or 48 h, then cells were collected for qPCR assay and IC50 detection using CCK-8.

For cells transfection, siRNAs target for AL590705.3, LINC02870, KDM4A-AS1, and MKLN1-AS were provided by Sangon (Shanghai, China). The control of siRNAs was named as si-NC. Detail sequences of siRNAs were showed in **Table 1**. Lipofectamine 3000 was used to transfected these siRNAs into Huh-7 cells following the instruction.

**Table 1:** The siRNAs sequences.

siRNA_id	Sense Sequence (5’-3’)	Antisense Sequence (5’-3’)
si-AL590705.3-1	GGUAGAAGCUCCUGUCUUAUC	UAAGACAGGAGCUUCUACCAA
si-AL590705.3-2	AGGUAAGUAGUUAGAUUAACU	UUAAUCUAACUACUUACCUGA
si-AL590705.3-3	AGUGCUUGUCAAUGAUCAACU	UUGAUCAUUGACAAGCACUCU
si-LINC02870-1	GCUUCCUGUUCACAGAGAAAC	UUCUCUGUGAACAGGAAGCUG
si-LINC02870-2	GAGAAACCUCAGACAAGAAGC	UUCUUGUCUGAGGUUUCUCUG
si-LINC02870-3	CGUGGUAGAUCAAGCCUCACA	UGAGGCUUGAUCUACCACGGG
si-KDM4A-AS1-1	GGUGGAUAUCAGAGAAUAAUA	UUAUUCUCUGAUAUCCACCAA
si-KDM4A-AS1-2	GGAGGUGCUUGGUCAACAAAG	UUGUUGACCAAGCACCUCCCG
si-KDM4A-AS1-3	CAAUAGAUUGAAUCAAGAAGU	UUCUUGAUUCAAUCUAUUGUG
si-MKLN1-AS-1	AGAUUUCUGUCCUGUGUUAAG	UAACACAGGACAGAAAUCUUG
si-MKLN1-AS-2	AGAGGAAAUUCAUAGAAUAGG	UAUUCUAUGAAUUUCCUCUUG
si-MKLN1-AS-3	GGUGGUGUUUCUCUCUGAAAG	UUCAGAGAGAAACACCACCAG

### qPCR and western bloting

The expression levels of AL590705.3, LINC02870, KDM4A-AS1, and MKLN1-AS were detected using qPCR method. Total RNA was isolated from Huh-7 cells using TriQuick Reagent. HiScript III RT SuperMix for qPCR (+gDNA wiper) was used to transcript total RNA to cDNA. ChamQ Universal SYBR qPCR Master Mix was used to evaluate the CT value. The relative expression was calculated using 2^−ΔΔ^CT. The primer sequences presented in **Table 2**.

**Table 2:** The primer sequences.

Primer Name	Sequence (5’-3’)
AL590705.3-F71	GCTCCTGTCTTATCAGGCCC
AL590705.3-R71	TTTCCTTGGCAGAACCACAA
LINC02870-F158	ACGTCGCCCATTTCTCATCA
LINC02870-R158	GAGGCTTGATCTACCACGGG
KDM4A-AS1-F80	AGGGTGAAAGGAACGTCCAC
KDM4A-AS1-R80	TGAAGTACTTTGCCAGGTCCC
MKLN1-AS-F84	CCGGGCCAATGTCCTATCTC
MKLN1-AS-R84	AAGCGCTTACACCTCAGACC
GAPDH-F131	GAGTCAACGGATTTGGTCGT
GAPDH-R131	GACAAGCTTCCCGTTCTCAG

Western blotting was used to determine FDX1 expression following the standard protocol. The primary antibodies information was FDX1 Polyclonal antibody (proteintech, #12592-1-AP), and beta actin recombinant antibody (proteintech, #81115-1-RR). Beta actin was the internal control.

### Evaluation the IC50 value and the ability of proliferation

Cells were inocated into 96-well plate with 8000 cells per well. After 24 h, cells were treated with elesclomol with different concentration (0, 0.1, 0.3, 1, 3, 10, 30, and 100 nM) for 48 h. CCK-8 regent (Servicebio, #G4103, 10 μL) was added into each well, and the OD value were detected after incubation 3 h. Graphpad prism was used to calculate the IC50 value.

After transfection for 48 h, Huh-7 cells (4000 cells per well) were used for CCK-8, and 800 cells used for clone formation to evaluate the ability of proliferation following standard protocol.

### Principal components analysis

To verify that the final gene effectively differentiates the HCC samples in the training cohort, test cohort, and entire cohort, principal component analysis (PCA) of the entire cohort and visualization of the results were performed using the “scatterplot3d” package. The PCA of the training cohort and test cohort and the visualization of the results were performed using the “Rtsne” and “ggPlot2” software packages.

### Functional gene set enrichment analysis

With a curated gene set (kegg.v7.5.1.symbols.gmt), gene set enrichment analyses (GSEA) software (https://www.gsea-msigdb.org/gsea/login.jsp) was applied to identify the significantly enriched pathways between the low- and high-risk groups based on the criteria p < 0.05 and FDR < 0.25.

### Immune infiltration analysis and potential drug prediction

ssGSEA was conducted to quantify the subgroup of tumor-infiltrating immune cells between the high- and low-risk groups and estimate their immune function. The ESTIMATE algorithm was used to evaluate the degree of infiltration of tumor cells and normal cells to calculate EstimateScore, ImmuneScore, and StromalScore. The prediction efficiency of immune checkpoint inhibitors may be related to the expression of immune checkpoint–related genes. Thus, the relationships between immune checkpoint genes and the cuproptosis-related signature were analyzed to investigate the potential role of CRDELSig and cuproptosis-related lncRNAs in immunotherapy for HCC. The potential response to immunotherapy of HCC patients was predicted using the Tumor Immune Dysfunction and Exclusion (TIDE) algorithm. Data from the Genomics of Drug Sensitivity in Cancer (GDSC) (https://www.cancerrxgene.org/) database were used to predict the response of HCC patients to chemotherapeutic drug therapy. The “pRRophetic” package was used to predict drug sensitivity by calculating the half-maximal inhibitory concentration (IC50) for each sample. IC50 indicates the effectiveness of a substance in inhibiting specific biological or biochemical processes.

### Statistical analysis

Statistical analysis was performed using R software (version 4.1.1). Overall survival analysis was performed using the Kaplan–Meier method and log-rank tests. Univariate and multivariate Cox regression analyses were performed to calculate the prognostic significance of lncRNAs in HCC patients. Pearson rank was used in the correlation analysis. Wilcoxon rank-sum test was used to analyze differences between two groups of quantitative data. The criterion for statistical significance was set at P <0.05.

## Results

### Construction and validation of a cuproptosis-related lncRNAs prognostic signature for HCC

**Figure 1:**
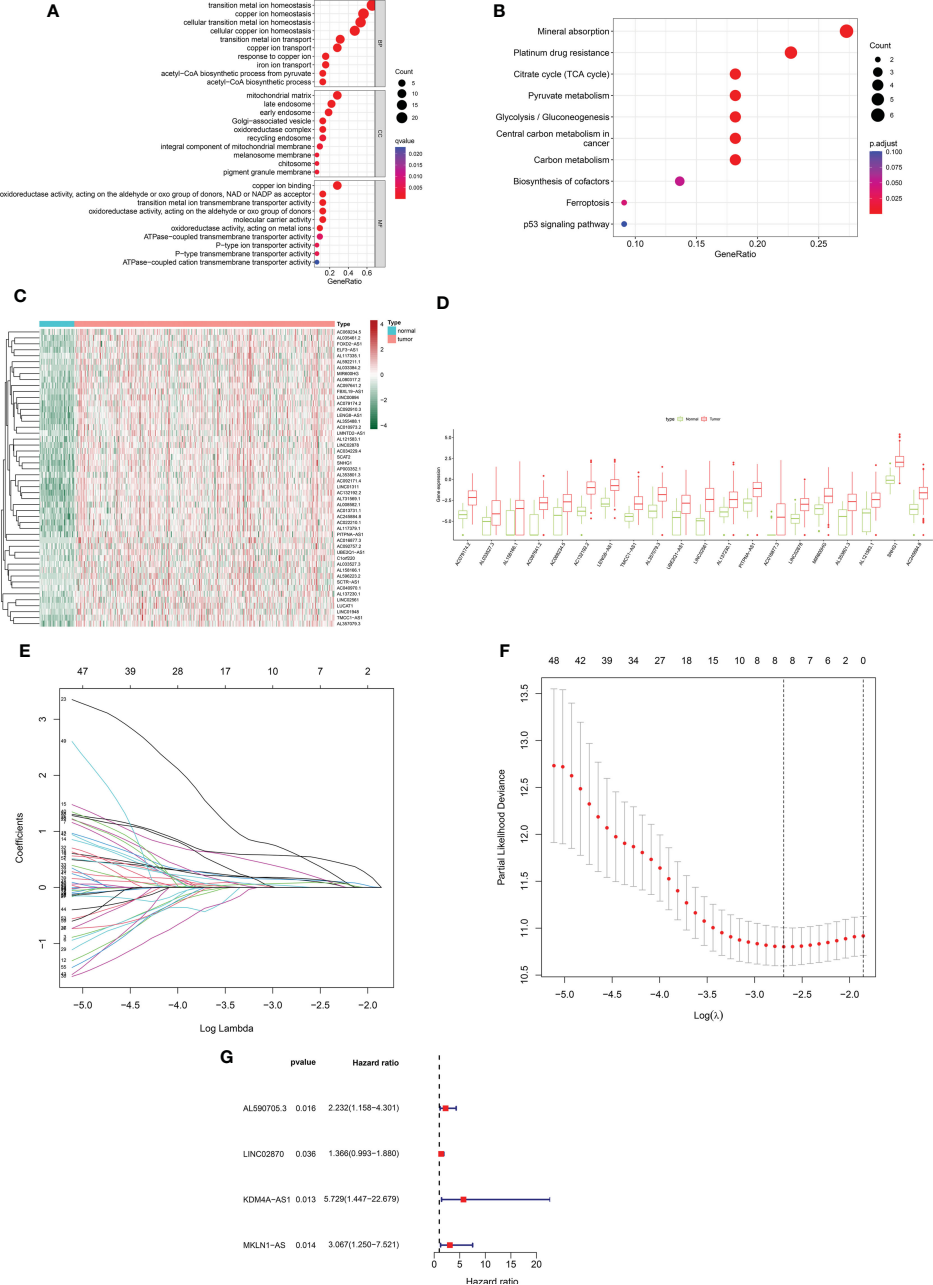
Construction of a prognostic risk profile for HCC patients based on cuproptosis-related lncRNAs in a training cohort. **(A)** GO enrichment analysis of cuproptosis genes. **(B)** KEGG enrichment analysis of copper death-related genes. **(C)** Heatmap of 50 cuproptosis-related differentially expressed lncRNAs in tumor and normal samples. **(D)** Boxplots of 20 cuproptosis-related differentially expressed lncRNAs in tumor and normal samples. **(E)** Distribution plot of the LASSO coefficient. **(F)** Distribution plot of the partial likelihood deviation of the LASSO regression. Eight variables were retained when the partial likelihood deviation reached the minimum (log lambda = –2.7). **(G)** The Forest plot showed 4 lncRNAs identified by multivariate Cox regression in the training cohort.

Based on 424 RNA-sequencing samples downloaded from TCGA, we extracted the expression matrix of 32 cuproptosis-related genes. Further functional enrichment analysis using the GO and pathway enrichment using the KEGG revealed (**Figure 1A**) that their biological process (BP) was mostly linked to copper ion transport, homeostasis, and other activities. The mitochondrial matrix, oxidoreductase complex, and other cellular components (CC) were primarily enriched, while molecular functions (MF) were mostly associated with functions like copper ion binding and oxidoreductase activity. In addition, KEGG was shown to be enriched in the mineral absorption, citrate cycle (TCA cycle), and other pathways (**Figure 1B**). This implied even more strongly that this gene set was closely linked to cuproptosis. Using Pearson correlation analysis (correlation coefficient > 0.5, P <0.05), we next discovered 572 cuproptosis-related lncRNAs. A total of 186 cuproptosis-related differentially expressed lncRNAs (CRDEL) were finally obtained for further analysis based on the analysis of differences between tumor and normal samples (log2|Fold Change| > 1, FDR <0.05). **Figures 1C, D** show the expression heat map of 50 differential lncRNAs and the box line plot of 20 differential lncRNAs.

**Figure 2:**
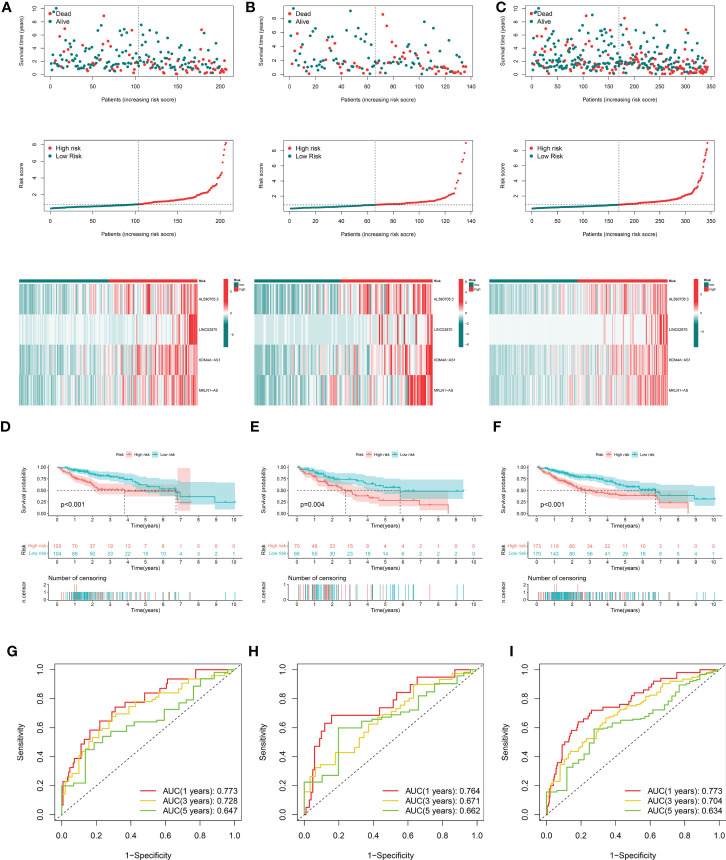
Evaluation and validation of cuproptosis-related lncRNA signatures for overall survival in patients with HCC in three datasets. Risk scores and expression profiles of seven-lncRNA signatures in the high- and low-risk groups showed in the training cohort **(A)**, testing cohort **(B)**, and entire cohort **(C)** Kaplan–Meier survival and ROC analyses in the training cohort **(D, G)**, testing cohort **(E, H)**, and entire cohort **(F, I)**, respectively.

After removing patients with survival duration of 30 days or less, as well as those with missing survival time and survival status information, a sample with 343 individuals with complete clinical survival information was obtained and integrated with the CRDEL expression matrix. A total of 343 cases were randomly divided into the training cohort (n = 207) and the test cohort (n = 136) in a 6:4 ratio. Subsequently, 59 lncRNAs significantly associated with HCC prognosis were identified in the training cohort using univariate Cox regression analysis (p < 0.05). After screening the variables using LASSO Cox regression analysis using the “lambda. min” criterion, eight prognosis-related lncRNAs were kept (**Figures 1E, F**). Multivariate Cox regression analyses identified four cuproptosis-related differentially expressed lncRNAs (CRDELSig, **Table 3**) that were all associated with poor HCC prognosis (HR(Hazard Ratio)> 1, **Figure 1G**). We computed the risk scores based on our prognostic traits using the following formula: Risk score = (0.8028 × expression of AL590705.3) + (0.3118 × expression of LINC02870) + (1.7455 × expression of KDM4A-AS1) + (1.1206 × expression of MKLN1-AS). Based on the median risk scores, risk models were built to categorize samples in the training and test sets into the high and low-risk groups. **Figure 2A** displays the distribution of risk scores and patients’ survival in the training cohort’s low- and high-risk groups. **Figures 2B, C** also display the test cohort as well as the complete cohort. The findings revealed that mortality was considerably higher in patients who surpassed the median risk score and were classified as “high risk.” In addition, the heat map showed that the characteristic lncRNAs were highly expressed in the high-risk group. Moreover, the Kaplan–Meier survival analysis revealed that in the training cohort, high-risk patients’ survival durations were considerably lower than those of low-risk patients (**Figure 2D**). Using the same risk score formula on the test cohort and the entire cohort yielded the same results (**Figures 2E, F**). Next, we built ROC curves to evaluate CRDELSig, and the ROC curves exhibited steady and robust predictive value for survival prediction at 1, 3, and 5 years for the training cohort, test cohort, and total cohort, with the training cohort AUC (1 year) of 0.773, AUC (3 years) of 0.728, and AUC (5 years) of 0.647 (**Figure 2G**); test cohort AUC (1 year) of 0.764, AUC (3 years) of 0.671, and AUC (5 years) of 0.662 (**Figure 2H**); and total cohort AUC (1 year) of 0.773, AUC (3 years) of 0.704, and AUC (5 years) of 0.634 (**Figure 2I**). All of these findings show that we were successful in developing the four cuproptosis-related lncRNA predictive signatures.

**Table 3:** The Multivariate Cox regression analyses identified four cuproptosis-related lncRNAs.

ID	COEF	HR	PVALUE
AL590705.3	0.8028	2.2317	0.0165
LINC02870	0.3118	1.3659	0.0356
KDM4A-AS1	1.7455	5.7289	0.0129
MKLN1-AS	1.1206	3.0667	0.0144

### Correlation of CRDELSig with clinicopathological features of HCC

**Figure 3:**
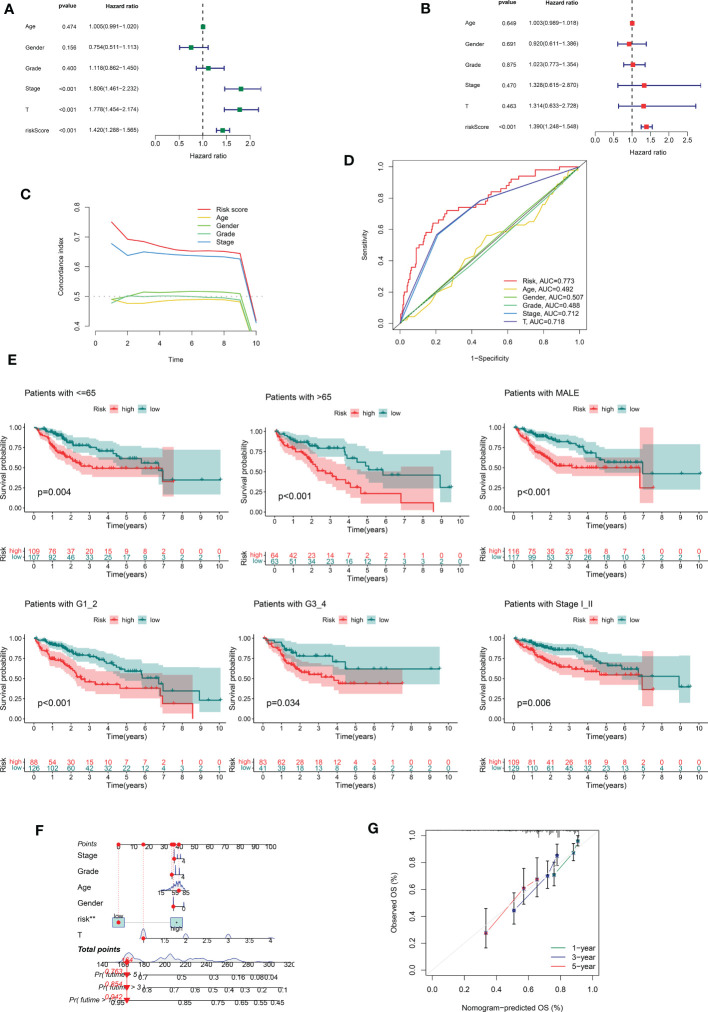
Correlations between risk score and different clinicopathological characteristics of HCC in the entire cohort. **(A)** Forest plot for univariate Cox regression analysis. **(B)** Forest plot for multivariate Cox regression analysis. **(C)** C-index for risk score. **(D)** ROC analysis of 1-year overall survival for multiple prognostic indicators of HCC samples. **(E)** Clinical stratification analysis of overall survival of patients with HCC in the high- and low-risk groups by age, gender, histological grade, and tumor stage. **(F)** Nomogram combining CRDELSig and clinicopathological characteristics for predicting prognosis of HCC patients in the training cohort. ROC analysis of the predictions of 1-, 3-, and 5-year survival by the nomogram in the training cohort. **(G)** Calibration curve analysis of the nomogram for the probability of overall survival at 1, 3, and 5 years.

In the following step, we used the risk scores to perform univariate and multivariate Cox regression analyses throughout the cohort. The HR shown in the univariate Cox regression analysis was 1.420, with 95% CI of 1288–1.565 (p < 0.001, **Figures 3A**) and the HR shown in the multivariate Cox regression analysis was 1.390, with 95% CI of 1.248–1.548 (p < 0.001, **Figure 3B**). These findings implied that the risk scores derived from the prognostic features were independent risk factors for HCC. The risk score also exceeded these standard clinicopathological characteristics in terms of the C-index curve, which was built in conjunction with age, gender, grading, and staging. The risk score was superior to this traditional clinicopathological profile (C-index). (**Figure 3C**). The ROC curve showed the same conclusion, with an area under the risk score curve of 0.773, which was better risk score greater than the comparably superior T-stage and Stage (**Figure 3D**). This implied that our prognostic feature model outperformed traditional clinicopathological variables in predicting HCC prognosis. Patients in the high-risk group had considerably worse prognosis than those in the low-risk group in all subgroups stratified according to clinical stratification analysis of the full TCGA cohort according to clinicopathological criteria such as age, gender, grade, and tumor stage (**Figure 3E**). In addition, using a combination of risk scores and independent clinical criteria, a nomogram was created to forecast the 5-year survival probability of HCC patients (**Figure 3F**). This is more conducive to individualized survival prediction in HCC patients. Calibration plots showed an excellent degree of agreement between the actual observations and nomogram projections in terms of 1-year, 3-year, and 5-year survival rates (**Figure 3G**).

**Figure 4:**
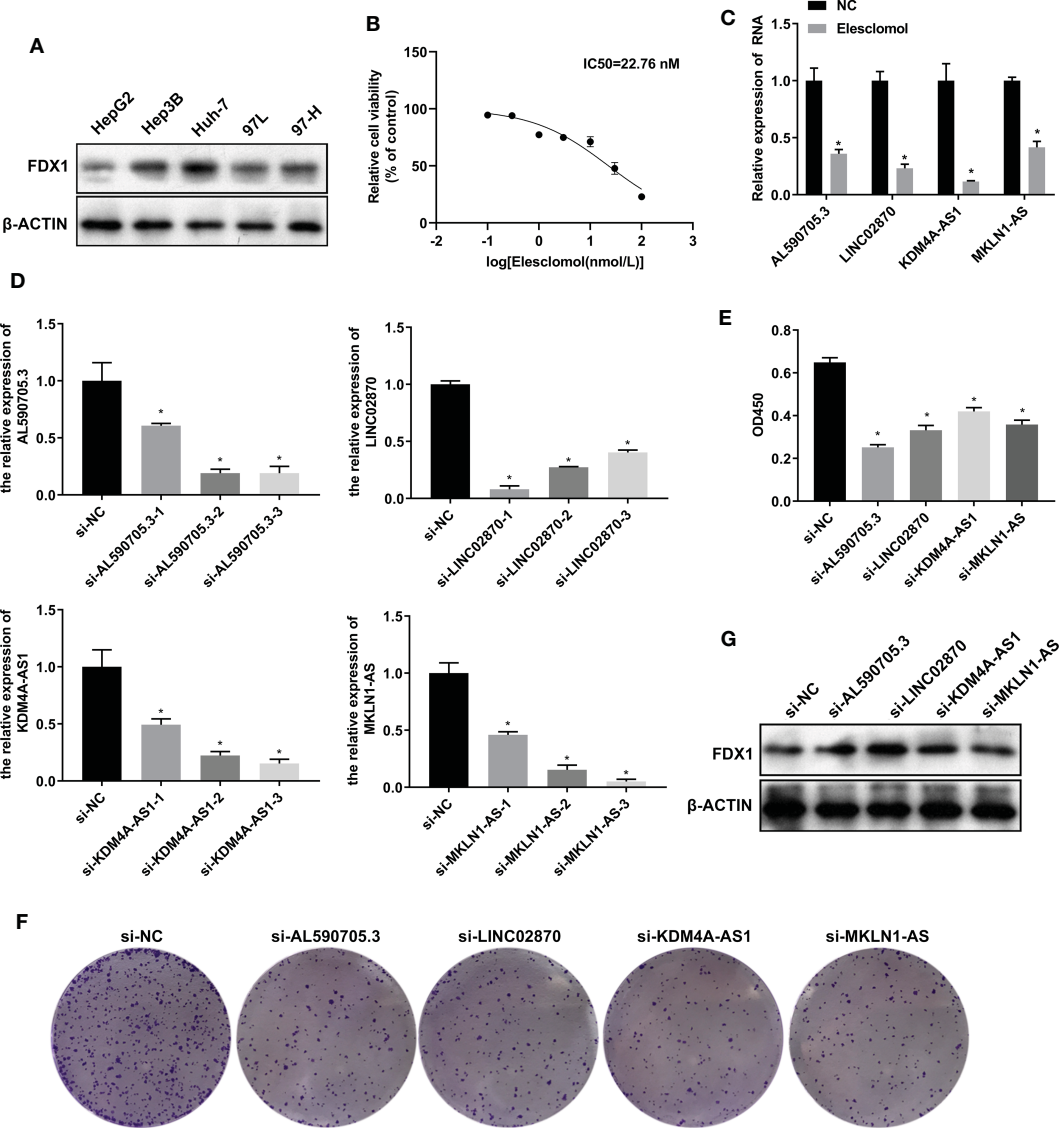
LncRNA AL590705.3, LINC02870, KDM4A-AS1, and MKLN1-AS participated in cuproptosis. **(A)** Westen blotting detected FDX1 expression in HepG2, Hep3B, Huh-7, 97L, and 97H cells. **(B)** CCK-8 was used to evaluated the IC50 value of elesclomol in Huh-7 cells after treated with elesclomol in different concentration (0, 0.1, 0.3, 1, 3, 10, 30, and 100 nM) for 48 h. **(C)** qPCR detected the expression levels of AL590705.3, LINC02870, KDM4A-AS1, and MKLN1-AS after treated with elesclomol for 24 h. **(D)** qPCR detected the interfere effective of siRNAs target for AL590705.3, LINC02870, KDM4A-AS1, and MKLN1-AS respectively. Huh-7 cells were transfected with siRNAs for 24 h, then cells were collected for qPCR analysis. **(E)** CCK-8 was used to evaluated the ability of proliferation. Huh-7 cells were transfected with siRNAs for 48 h, and then CCK-8 was conducted. **(F)** Clone formation was used to evaluated the ability of proliferation. Huh-7 cells were transfected with siRNAs for 48 h, and then clone formation was conducted. **(G)** Western blotting detected the FDX1 expression in transfected with siRNAs for 48 h Huh-7 cells. *, P value less than 0.05.

To investigate the role of AL590705.3, LINC02870, KDM4A-AS1, and MKLN1-AS in liver cancer cells, we fistly detected the expression of FDX1 in HepG2, Hep3B, Huh-7, 97L, and 97H cells. Results showed that FDX1 was high expression in Huh-7, relative to HepG2, Hep3B, 97L, and 97H cells, indicating that Huh7 had more sensitive to cuproptosis (**Figure 4A**). Therefore, Huh-7 was selected for further study. To obtain the appropriate treatment concentration of elesclomol, Huh-7 was treated with elesclomol in different concentration (0, 0.1, 0.3, 1, 3, 10, 30, and 100 nM) for 48 h, and then analyzed the cell viability. As showed in **Figure 4B**, Huh-7 cells viability was decreased with the increase of elesclomol, and the IC50 was 22.76 nM. So 10 nM of elesclomol, about 1/2 of IC50 value, was selected for further experiments. After treatment with elesclomol for 24 h, the expression levels of AL590705.3, LINC02870, KDM4A-AS1, and MKLN1-AS were all decreased relative to respective control (**Figure 4C**). To investigate the function of these lncRNAs, siRNAs target for these RNA was transfected into Huh-7 cells for 24 h, then the interfere efficiency of siRNAs were evaluated through qPCR. As presented in **Figure 4D**, all siRNAs target for RNA had interfere effect relative to respective control, and si-AL590705.3-2, si-LINC02870-1, si-KDM4A-AS1-3, and si-MKLN1-AS-3 had the best effect on respective other siRNAs, which were selected for next study. After transfection with siRNAs for 48 h, Huh-7 cells proliferation were inhibited relative the si-NC group (**Figures 4E, F**). Additionally, inhibition the expression levels of AL590705.3, LINC02870, KDM4A-AS1, and MKLN1-AS increased FDX1 expression in Huh-7 cells (**Figure 4G**). The above results confirmed that AL590705.3, LINC02870, KDM4A-AS1, and MKLN1-AS participate in the progress of cuproptosis.

### PCA

**Figure 5:**
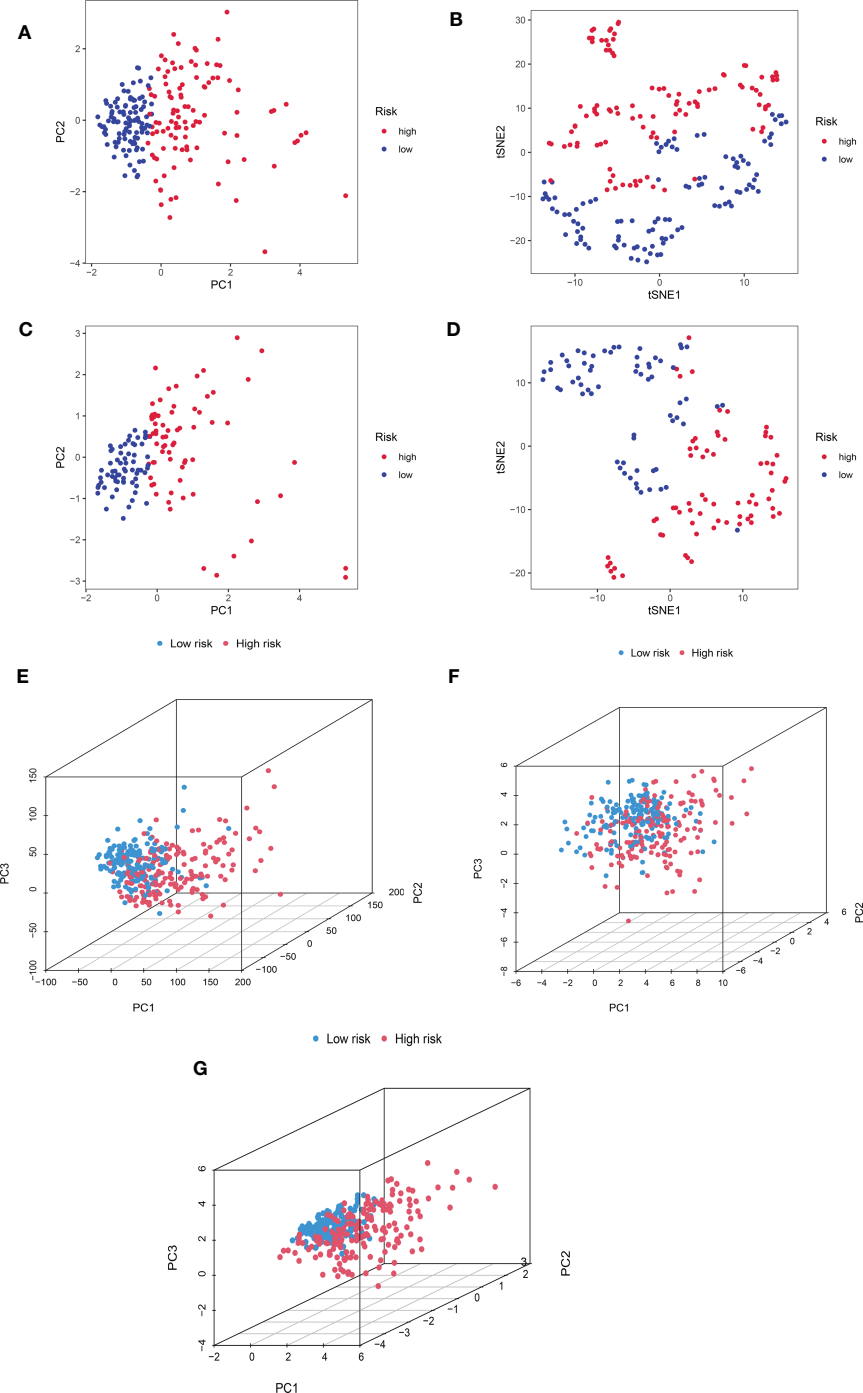
Principal component analysis. **(A)** PCA for the training cohort. **(B)** t-sne of the training cohort. **(C)** PCA for the test cohort. **(D)** t-sne of the test cohort. **(E)** PCA 3D map based on whole gene expression profiles, **(F)** PCA 3D map based on fever-related genes, **(G)** PCA 3D map based on fever-related differential expression of lncRNAs in TCGA entire cohort.

We additionally carried out a PCA on the training cohort using the “Rtne” package to better reflect the capability of CRDELSig to distinguish samples. The results are shown in **Figure 5A**. Specifically, the PCA of the training cohort indicated that the high-risk and low-risk samples were clustered separately. The t-SNE(t-distribution stochastic neighborhood embedding)of the training cohort demonstrated similar results (**Figure 5B**). Applying CRDELSig to the test cohort for PCA, the results were also consistent (**Figures 5C, D**). In order to show differences between the high- and low-risk groups, we pooled the two cohorts and performed PCA based on the total gene expression profile, 32 cuproptosis-related genes, and 186 cuproptosis-related lncRNAs. The different distribution states for the high and low-risk categories are all depicted in **Figures 5E–G**. These findings imply that CRDELSig can more effectively distinguish between groups at high and low risk.

### Association of CRDELSig with the tumor immune microenvironment

**Figure 6:**
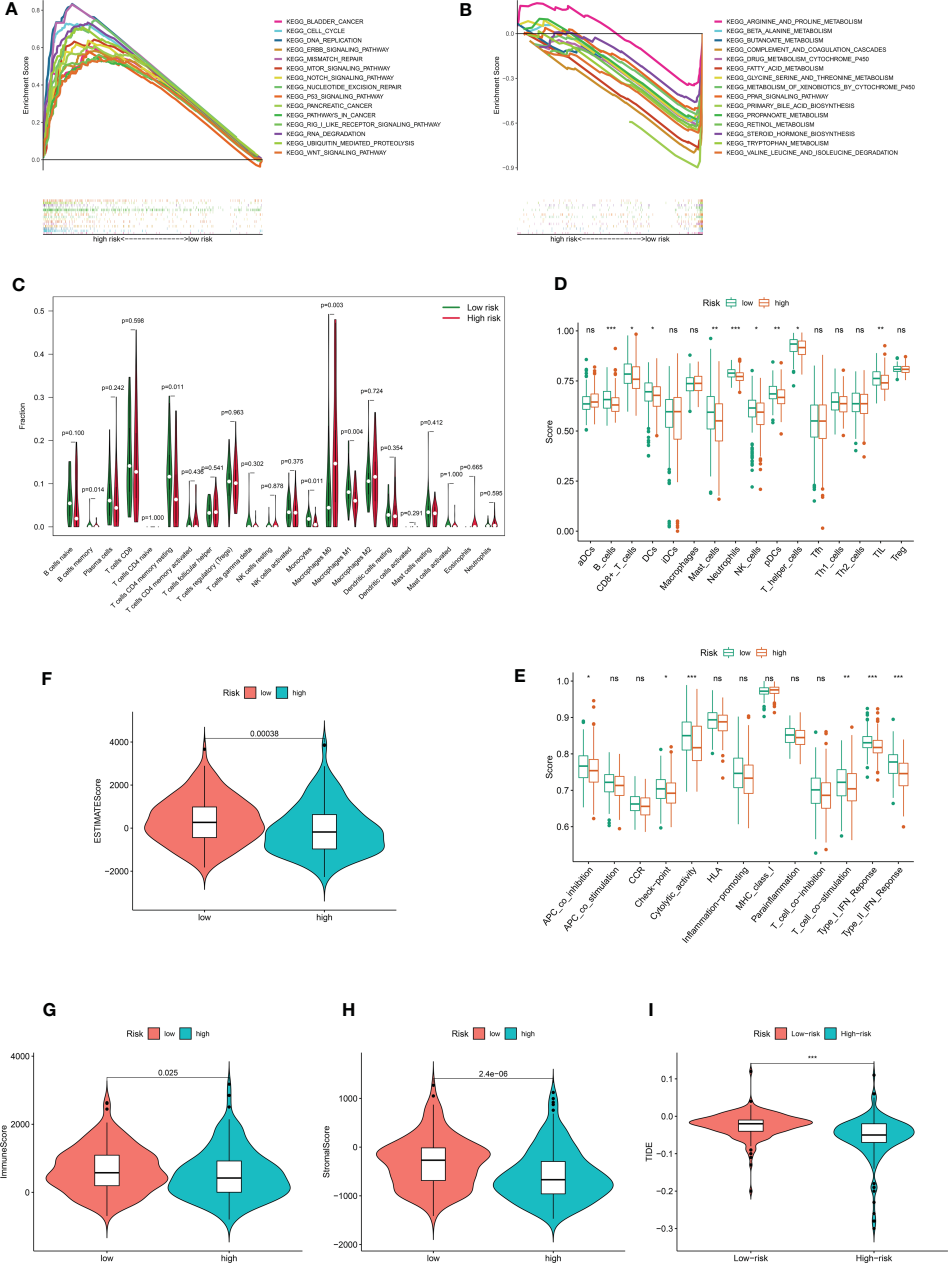
The immune landscape between high- and low risk- groups. Demonstrates that 15 representative KEGG pathways were significantly enriched in **(A)** high risk and **(B)** low risk groups, respectively. **(C)** Violin plot of 22 immune cell infiltrations based on the CIBERSORT algorithm. Immune cell scores **(D)** and immune function scores **(E)** in high- and low-risk groups based on ssGSEA algorithm. Comparison of estimate scores**(F)**, immune scores**(G)**, and stromal scores**(H)** between HCC patients in high- and low-risk groups. **(I)** Comparison of TIDE prediction scores between the high- and low-risk groups in the TCGA_LIHC dataset. *p<0.05, **p<0.01, ***p<0.001. no significant, p>0.05.

The underlying risk mechanism that produces disparities between low- and high-risk samples was further investigated using GSEA. The findings demonstrated that the high-risk group was enriched for several processes linked to the progression of tumor proliferation, including the cell cycle, the ERBB signaling pathway, mismatch repair, the p53 signaling pathway, ubiquitin-mediated protein hydrolysis, and the WNT signaling pathway (**Figure 6A**). Additionally, we discovered that CRDELSig was linked to pancreatic and bladder cancer. In the high-risk group, immune-related pathways such as the mTOR signaling pathway and the NOCTH signaling pathway were also enriched. The low-risk group, however, was primarily enriched for pathways associated with metabolism, including those for arginine and proline metabolism, beta-alanine metabolism, bile acid metabolism, and drug metabolism cytochrome p450 (**Figure 6B**). This might be one of the causes of the two groups’ differential prognoses.

In light of the GSEA data, we conducted additional investigation into the variations in the tumor immune microenvironment between the two groups. The extent of infiltration of 22 immune cells was explored using the CIBERSORT algorithm. The results showed significant differences in the infiltration of five immune cell types (including B cells memory, T cells CD4 memory resting, monocytes, macrophages M0, and macrophages M1) between in the high and low-risk groups (**Figure 6C**). Interestingly, antitumor M1 macrophages predominately infiltrated the low-risk group of HCC samples, while proliferative M0 macrophages with tumor-promoting growth predominantly infiltrated the high-risk group of HCC samples. All other differentially infiltrating cells were less abundant in the high-risk group. The ssGSEA algorithm was then utilized to investigate the immune infiltration landscape (infiltration landscape) between the high- and low-risk groups, with each typical immune cell and immune function scored, as shown in **Figures 6D, E**. B_cells, CD8+_T_cells, DCs Dendritic cells, Mast_cells, Neutrophils, NK_cells, pDCs, T_helper_cells, and TIL showed significant differences in infiltration between the two groups. Interestingly, consistent with the CIBERSORT algorithm, all of these immune cells were less abundant in the high-risk group of HCC samples. Additionally, there was a substantial difference between the high-risk and low-risk groups in terms of immune functions such as APC Antigen Presenting Cell(APC)co-inhibition, checkpoint, cytolytic activity, T-cell co-simulation, type I IFN response, and type II IFN response. The high-risk group showed decreased levels of each of these immunological functions. All of these findings suggest an immunosuppressed tumor immunological microenvironment in the high-risk group of HCC patients, which could be an additional factor contributing to the generally poor prognosis of this patient population. Then, for each sample in both groups, we analyzed the tumor microenvironment and related traits, such as EstimateScore, ImmuneScore, and StromalScore. The findings demonstrated that, compared with the low-risk group, patients with HCC in the high-risk group had considerably lower stromal, immunological, and estimating scores (**Figures 6F–H**). Additionally, we examined whether CRDELSig could forecast the advantages of immunotherapy in both groups using the TIDE algorithm, which was created to predict response to immunotherapy. Patients in the high-risk group had significantly lower TIDE scores than those in the low-risk group (**Figure 6I**), indicating that high-risk patients responded better to immunotherapy. These findings imply that the tumor immune microenvironment differs between the high and low-risk groups, that patients in the high-risk group are immunocompromised with relatively low tumor clearance, and that HCC is more likely to proliferate and progress in the high-risk group, resulting in poor outcomes.

### Immune checkpoint analysis and prediction of sensitive drugs

**Figure 7:**
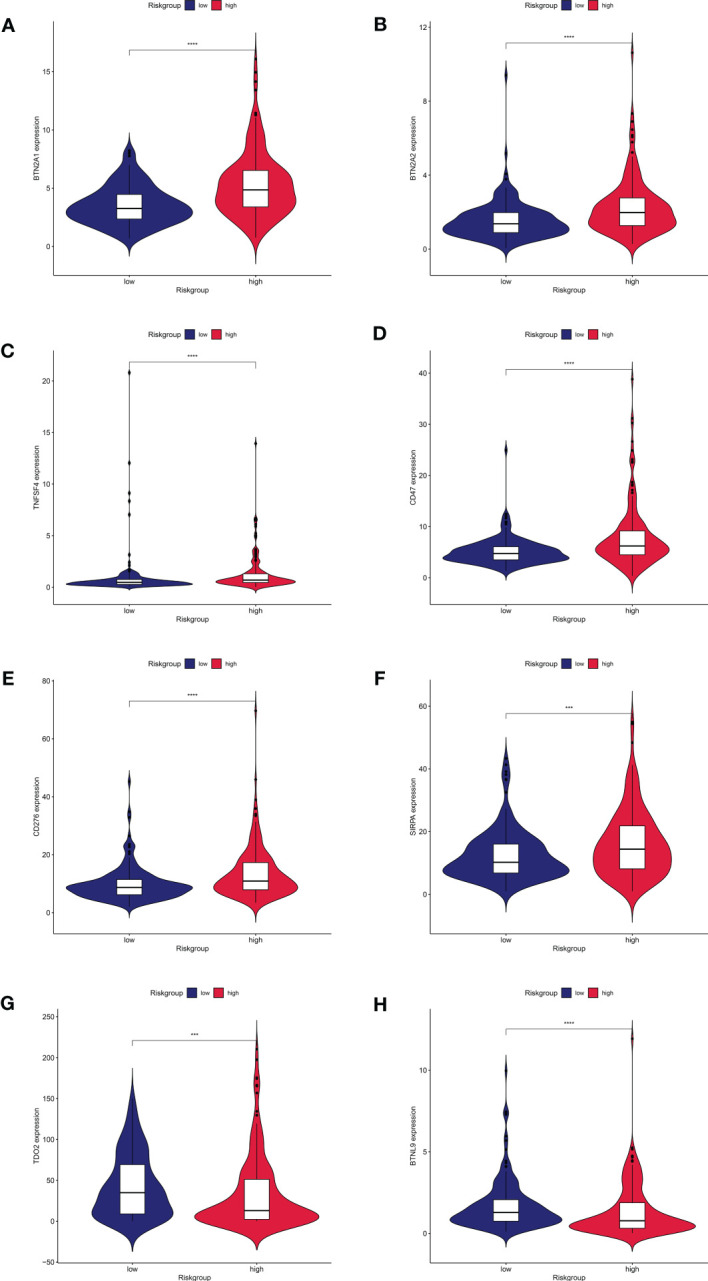
Expression of immune checkpoint genes between high and low risk groups.8 immune checkpoint genes were significantly different in high- and low risk- groups. The expression of **(A)** BTN2A1, **(B)** BTN2A2, **(C)**TNFSF4, **(D)**CD47, **(E)**CD276, **(F)**SIRPA, **(G)**TDO2, and **(H)**BTNL9 showed significant differences in the high and low risk groups.

Distribution of immune checkpoint genes was examined after the investigation of immune function. BTN2A1, BTN2A2, TNFSF4, CD47, CD276, and SIRPA (**Figures 7A–F**) were comparatively highly expressed in the high-risk group, but TDO2 and BTNL9 (**Figures 7G, H**) were relatively lowly expressed in the high-risk group, according to our analysis of the expression profiles of two groups of immune checkpoint genes. We predicted the potentially sensitive medications for both groups based on the drug IC50 supplied by the Genomics of Therapeutic Sensitivity in Cancer (GDSC) (https://www.cancerrxgene.org/) to tailor drug therapy for the high- and low-risk groups. In the high-risk group, paclitaxel and gemcitabine exhibited a lower IC50 and higher sensitivity (**Supplemental Figures 1A and B**). The low-risk group, however, showed lower IC50 and higher sensitivity for elesclomol, methotrexate, rapamycin, and sorafenib (**Supplemental Figures 1C–F**). Sorafenib has been demonstrated to dramatically increase patients’ overall survival and is currently the first-line drug in chemotherapy for HCC. Elesclomol is an effective copper ion carrier that causes cancer cells to undergo apoptosis by promoting the copper death process, resulting in an antitumor effect. These findings could help develop personalized immunotherapy and targeted treatments for HCC patients.

### Tumor mutation burden and correlation analysis

**Figure 8:**
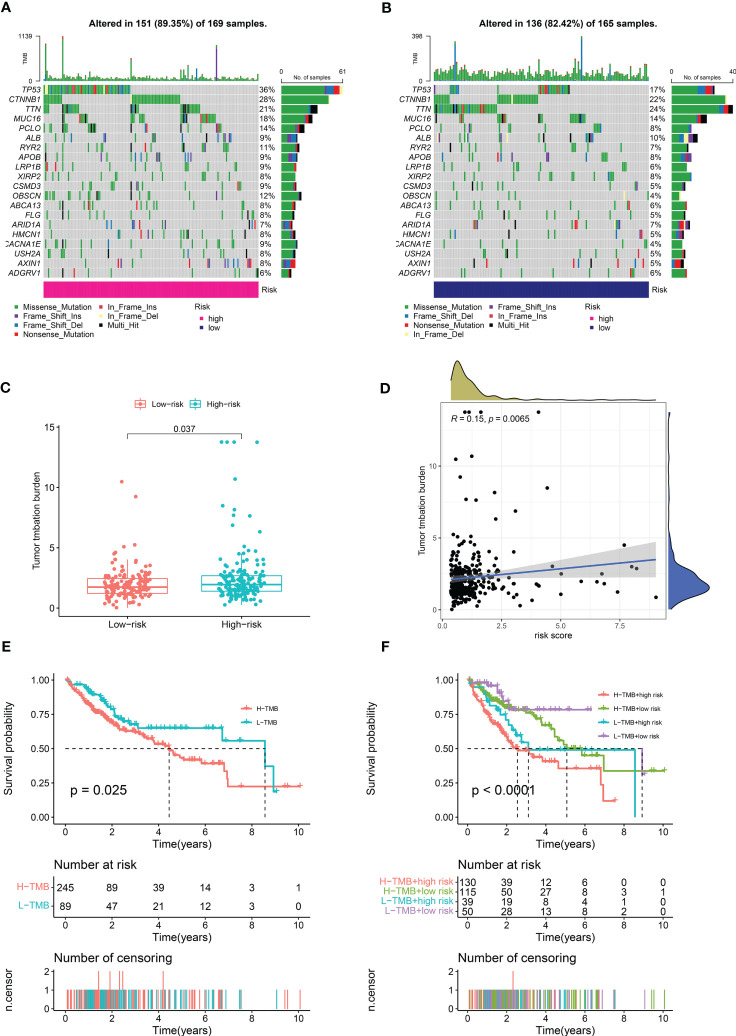
Tumor Mutational Burden and related analysis. Waterfall plots of mutated genes in **(A)** the high-risk group and **(B)** the low-risk group. **(C)** TMB difference in the high- and low-risk patients. **(D)** Correlation point plot for risk score and TMB. **(E)** Kaplan–Meier curve analysis of OS is shown for patients in high and low TMB. **(F)** Kaplan–Meier curve analysis of OS is shown for patients classified according to the TMB and cuproptosis-related lncRNA model.

We calculated the mutations in each group based on the TMB scores generated from the TCGA somatic mutation data to determine the differences in cancer-associated mutations between the high-risk and low-risk groups, and discovered that the TMB was higher in the high-risk group than in the low-risk group (**Figures 8A, B**). TP53, CTNNB1, TTN, MUC16, and PCLO had the top five mutation frequencies in the high-risk group, while CTNNB1, TTN, TP53, MUC16, and ALB had the top five mutation frequencies in the low-risk group. Anticancer genes, such as TP53, had a higher mutation frequency in the high-risk group (36% *vs*. 17%), whereas oncogenes such as CTNNB1 had a higher mutation frequency in the low-risk group (28% *vs*. 21%). Furthermore, we investigated the relationship between the risk scores calculated by CRDELSig and tumor mutation load and discovered that the risk scores were substantially associated with TMB (R = 0.15, p < 0.05, **Figures 8C, D**). Based on the TMB score, we then separated HCC patients into high- and low-mutation groups. Kaplan–Meier survival analysis showed relatively low survival rates in the high-mutation group of HCC patients (**Figure 8E**). We subsequently analyzed the two various environmentally distinct subgroups to ascertain whether CRDELSig was a more accurate predictor of survival than TMB. Patients with high risk and high TMB had the worst prognosis, as illustrated in **Figure 8F**, whereas those with low risk and low TMB had the best prognosis. Additionally, the prognosis of low risk plus high TMB was superior to high risk plus low TMB. These findings undoubtedly imply that CRDELSig has superior prognosis prediction potential compared with TMB.

## Discussion

HCC is an insidious malignancy, and therefore, the majority of patients receive diagnosis at an advanced stage, usually with poor prognosis and a serious impact on human health (25). Recent studies have suggested a new form of cell death—cuproptosis. Copper deposition promotes lapidated protein aggregation and the destabilization of Fe–S cluster proteins, culminating in proteotoxic stress and, eventually, cell death (16). Tumors are intimately related to cuproptosis brought on by an imbalance in copper ions. And the prognostic signature of cuproptosis-related genes showed an excellent potential for predicting the prognosis of tumor (26). Moreover, cuproptosis were reported correlated with tumor immune microenvironment in many tumors, such as renal cancer, breast cancer, bladder carcinomas, HCC, and etc (27–30). For example, cuproptosis was negative correlated with tumor-infiltrating lymphocytes, and showed a noninflamed tumor microenvironment phenotype on the single cell level with low sensitive to immunotherapy in patient with bladder carcinomas (29). In addition, Zhang et al. showed that the increase of protumor immune infiltrates and highly expressed immune checkpoints in HCC patients with high cuproptosis-related risk score (31). Another study also indicated that cuproptosis-related genes were significantly correlated with immune cell infiltration, anti-tumor drug sensitivity, and tumor mutational burden in patient with HCC (30). Their research, however, did not show whether prognostic characteristics of HCC tumors were related with the immune microenvironment. The tumor immune microenvironment is important in cancer immunosuppression, which leads to tumorigenesis and tumor growth, as well as resistance to chemotherapy and immunotherapy. As a result, it is crucial to study whether cuproptosis-related lncRNAs are linked to differences in HCC tumor immune microenvironment. Furthermore, due to differing analytical methodologies and study strategies, additional cuproptosis-related lncRNAs may be more closely associated with HCC prognosis. Therefore, further research on copper death–related lncRNAs is warranted.

In this study, we identified copper death–associated lncRNAs by Pearson correlation analysis of 10 copper death genes and 22 copper metabolism–associated genes. Subsequently, lncRNAs differentially expressed in cancerous and normal tissues were analyzed. Four copper death–related lncRNA prognostic features (CRDELSig) were finally constructed by univariate Cox analysis, LASSO regression, and multivariate Cox analysis to predict the prognosis of HCC patients. The Kaplan–Meier survival analysis revealed that there were substantial differences in survival periods between high-risk and low-risk individuals as determined by CRDELSig. PCA demonstrated that CRDELSig was able to efficiently distinguish between high- and low-risk patients. Furthermore, the clinical stratification study revealed that CRDELSig was associated with the clinicopathological characteristics. Univariate and multifactorial Cox analyses demonstrated that our prognostic signature could be used as an independent prognostic risk factor for patients with HCC. Therefore, this signature was used as a prognostic risk model to predict survival of HCC patients. The ROC curves generated using data from the entire cohort, the test cohort, and the training cohort demonstrated the validity and reliability of our risk profile prediction capacity. Nomogram was able to provide clinical diagnostic and treatment recommendations by visually predicting the 1-, 3-, and 5-year survival rates of individual HCC patients.

We identified four lncRNAs, namely AL590705.3, LINC02870, MKLN1-AS, and KDM4A-AS1. In Huh-7 cells, more sensitive to elesclomol, these four lncRNA expression were downregulated after treated by elesclomol. And inhibition the expression of AL590705.3, LINC02870, MKLN1-AS, and KDM4A-AS1 decreased the ability of proliferation but increased FDX1 expression, indicating that AL590705.3, LINC02870, MKLN1-AS, and KDM4A-AS1 participated in the progression of cuproptosis. Additionally, these four lncRNAs’ high expression in the high-risk group was associated with poor prognosis. Previous research has linked MKLN1-AS overexpression to a worse prognosis in patients with HCC (32). MKLN1-AS promotes HCC cell proliferation, migration, and invasion by targeting and stabilizing YAP1 mRNA, and it also stimulates YAP1 expression *in vivo*, resulting in HCC (33, 34). AL590705.3 is a key component of the ferroptosis-associated lncRNA predictive model for gastric cancer (35). LINC02870 was involved in the development of another HCC prognostic model (36), but its function is unknown. KDM4A-AS1 is aberrantly overexpressed as an oncogene in HCC and promotes HCC growth and metastasis through the miR-411-5p/KPNA2/AKT axis, and high KMD4A-AS1 levels are associated with poor clinical features and poor prognosis (37). Furthermore, KDM4A-AS1, a tumor promoter, was considerably enhanced in both cell lines and cancer tissues in castration-resistant prostate cancer (CRPC), whereas KDM4A-AS1 deletion dramatically lowered cell viability, *in vitro* proliferation, migration, and *in vivo* tumor growth (38). To summarize, all four essential genes influence metabolism-related pathways and have a role in tumor development and progression; our study was first time to find that these four lncRNAs participated in the development of HCC cuproptosis.

We conducted GSEA analysis to investigate the putative molecular mechanisms of CRDELSig in the course of HCC. According to our findings, the cell cycle, ERBB signaling pathway, mismatch repair, p53 signaling pathway, ubiquitin-mediated protein hydrolysis, WNT signaling pathway, and various other processes associated with tumor proliferation and progression may contribute to the high-risk group’s poor prognosis. According to one study, the lncRNAs HERH-1 and HERH-4 increase the expression of cell cycle protein A2 to speed up the cell cycle of advanced HCC (39). Furthermore, overexpression of EVA1 promotes HCC cell growth, invasion, and migration *in vitro via* the ERBB–PI3K–AKT pathway (40). LINC00662 activates the Wnt/β-catenin signaling pathway in HCC cells in an autocrine manner by inducing WNT3A secretion, further promoting HCC cell proliferation, cell cycle, and tumor cell invasion, while inhibiting HCC cell apoptosis (41). Additionally, we discovered that immune-related pathways such as mTOR and NOCTH were enriched in the high-risk groups. However, the immunological escape of HCC tumors is significantly aided by the ERBB signaling pathway (42). In contrast, the low-risk group was mainly enriched in metabolism-related pathways. These results provide further evidence for the association of cuproptosis with the differences in tumor immune microenvironment characteristics between the two groups, which may serve as new therapeutic targets. Hence, better knowledge of the connections between CRDELSig and tumor immune modulation is critical.

To further analyze the differences in the tumor immune microenvironment between the two groups, we explored the extent of infiltration of 22 immune cell types using the CIBERSORT algorithm. According to the findings, M0 macrophages primarily invaded the high-risk group of HCC samples, whereas M1 macrophages primarily infiltrated the low-risk group of HCC samples. Multiple cytokines are produced by M1 macrophages, which also express major histocompatibility complexes and destroy tumor cells (43, 44). Tumor-associated macrophages play an important role in the tumor microenvironment, participating in angiogenesis, extracellular matrix (ECM) remodeling, cancer cell proliferation, metastasis, and immunosuppression, as well as resistance to chemotherapeutic agents and immunotherapy, such as checkpoint blockade (44, 45). In contrast, adequately activated macrophages could promote cancer cell phagocytosis and eliminate cytotoxic tumors (44). Therefore, tumor immunity is critical in the therapy of tumors. The ssGSEA algorithm then evaluated the immune infiltration landscape between the high- and low-risk groups, revealing substantial variations in typical immune cell infiltration between the two groups, with both being less infiltrated in HCC samples from the high-risk group. Furthermore, the immunological function of the high-risk group was impaired. These findings imply that the tumor immune microenvironment in high-risk HCC patients is immunosuppressive. According to previous research, pyroptosis-mediated tumor clearance is accomplished through increased immune activation and function, and decrease in CD4+ and CD8+ cell populations hinders tumor regression (46). The difference in immune infiltration between the high- and low-risk groups, similar to pyroptosis, may be because tumor cells in the high-risk group samples inhibit immune activation induced by copper death to sustain their development. In contrast, immune cell infiltration and immune function were more plentiful and active in samples from the low-risk group, which contributed to HCC tumor regression. Moreover, ESTIMATEScore, ImmuneScore, and StromalScore were lower in the high-risk group, indicating a lesser infiltration of immune cells, more stromal cells, and higher tumor purity in the high-risk group samples. This could also explain the apparent difference in prognosis between the two groups.

Tumor cells upregulate negative stimulatory chemicals to boost intrinsic tumor activity or change their surrounding tumor microenvironment to modify immunological checkpoints, increasing immune evasion and encouraging tumor progression and metastasis (9). There has been a significant improvement in the outcome of solid malignancies such as malignant melanoma, lung cancer, and kidney cancer with the use of Immune checkpoint inhibitors(ICIs)in the treatment of a wide spectrum of tumors (47–50). Therefore, we further explored the distribution of immune checkpoint genes in the high- and low-risk groups, and found that BTN2A1, BTN2A2, TNFSF4, CD47, CD276, and SIRPA were highly expressed in the high-risk group. Wang et al. found that blocking CD47 promoted antitumor immunity *via* the CD103+ dendritic cell–NK cell axis in a mouse model of HCC, thereby inhibiting HCC proliferation progression (51). Secondary SIRPA metastasis enhanced the development of mouse HCC cells *in vivo* by changing the inflammatory milieu and promoting angiogenesis (52). These findings imply that elevated expression of immune checkpoint genes in high-risk HCC patients may be linked to tumorigenesis. However, high-risk HCC patients had considerably lower TIDE scores, indicating a greater response to immunotherapy. In the high-risk group, paclitaxel and gemcitabine showed lower IC50 and increased sensitivity. However, in the low-risk group, elesclomol, metheotrexate, rapamycin, and sorafenib showed lower IC50 and higher sensitivity. Recurrent tumor models were reported to be inhibited by gemcitabine and alphaPD-1 blockers, with decreased tumor sizes and increased numbers of tumor-free animals (53). Elesclomol is a copper ion carrier, and while clinical studies for its use as a cancer treatment agent have been unsuccessful, subsequent research has revealed that it can aid patients whose tumors rely on mitochondria for energy production (54). Furthermore, elesclomol could be utilized to treat a variety of tumors that are particularly susceptible to the cuproptosis process, such as cancer cells with high FDX1 expression (16). Sorafenib has helped many HCC patients in the clinical setting. These findings could have a significant impact on customized immunotherapy and targeted therapy for patients with HCC.

Due to the relative rise in neoantigens in tumors with high TMB, intra-tumoral immunotherapy may be more successful in these patients (55–57). The TMB of patients in the high-risk group was much higher than that in the low-risk group, according to our analysis of the patients’ mutation data, indicating that immunotherapy may be more beneficial for patients in the high-risk group. In the high-risk group, we discovered that TP53 was most frequently mutated, whereas in the low-risk group, CTNNB1 was most frequently mutated. A common mutation in HCC is in the tumor suppressor gene TP53, sometimes called P53. Tumor differentiation, vascular invasion, serum methemoglobin levels, and tumor staging are all impacted by TP53 mutations (58, 59). A number of missense mutations within the TP53 gene’s DNA binding domain are tumor-promoting (60). Mutant TP53 promotes the formation of M2-like macrophages that promote tumor growth, while inhibiting the infiltration of cytotoxic T cells and NK cells into the tumor (61). *In vitro* and *in vivo*, p53 prevents the creation of cholesterol, which slows the growth of tumors (62). Immune evasion and anti-PD-1 resistance are promoted by CTNNB1 (63). These findings imply that immunosuppression in the high-risk group is related to a higher frequency of TP53 mutations. Furthermore, we investigated the relationship between the risk scores based on CRDELSig calculations and tumor mutation load, and discovered that the risk scores were substantially associated with TMB. It also implies that high-risk patients may be more active in their response to immunotherapy. Moreover, in the survival analysis combining the risk score and TMB, the risk score revealed better correlation with prognosis. This implies that CRDELSig, rather than TMB, is a better predictor of survival in HCC patients.

This research has certain limitations. First, further basic research is required to validate the mechanism of our screened lncRNAs in cuproptosis. Second, no external validation of the characteristics was undertaken because we were unable to find datasets having the four lncRNAs expression levels, clinical features, and survival status of HCC patients. Our research serves as the foundation for more in-depth research.

Overall, we succeeded in creating a signature of cuproptosis-related lncRNAs that predicts survival time in HCC patients with high specificity and sensitivity. The nomogram constructed using age, clinical TNM staging, and risk score of the four cuproptosis -associated lncRNAs can be a simple tool for predicting survival time in HCC patients. More importantly, our findings imply that copper death–associated lncRNAs are related to the tumor immune microenvironment of HCC and even the efficacy of immunotherapy, providing a theoretical foundation for future research.

## Data availability statement

The datasets presented in this study can be found in online repositories. The names of the repository/repositories and accession number(s) can be found in the article/**Supplementary Material**.

## Author contributions

B-LW: design of the work and revising manuscript critically for important intellectual content. WY, J-HX, J-SZ, B-LM, and P-ZW: acquisition, analysis, and interpretation of data for the work. WY, J-HX, and J-SZ: drafting the work. All authors contributed to the article and approved the submitted version.

## Supplementary material

Supplementary Figure 1 Potential drug prediction. Boxplot showing the mean differences in estimated IC50 values of 6 representative drugs. **(A)**paclitaxel, **(B)**gemcitabine, **(C)**Elesclomol, **(D)**Methotrexate, **(E)**Rapamycin, and **(F)**Sorafenib.
