# Supplementary figures and images for: Identification of a cuproptosis and copper metabolism gene–related lncRNAs prognostic signature associated with clinical and immunological characteristics of hepatocellular carcinoma
Source: Front Oncol. 2023 Mar 28;13:1153353. doi: 10.3389/fonc.2023.1153353 (PMC10086263; doi:10.3389/fonc.2023.1153353)

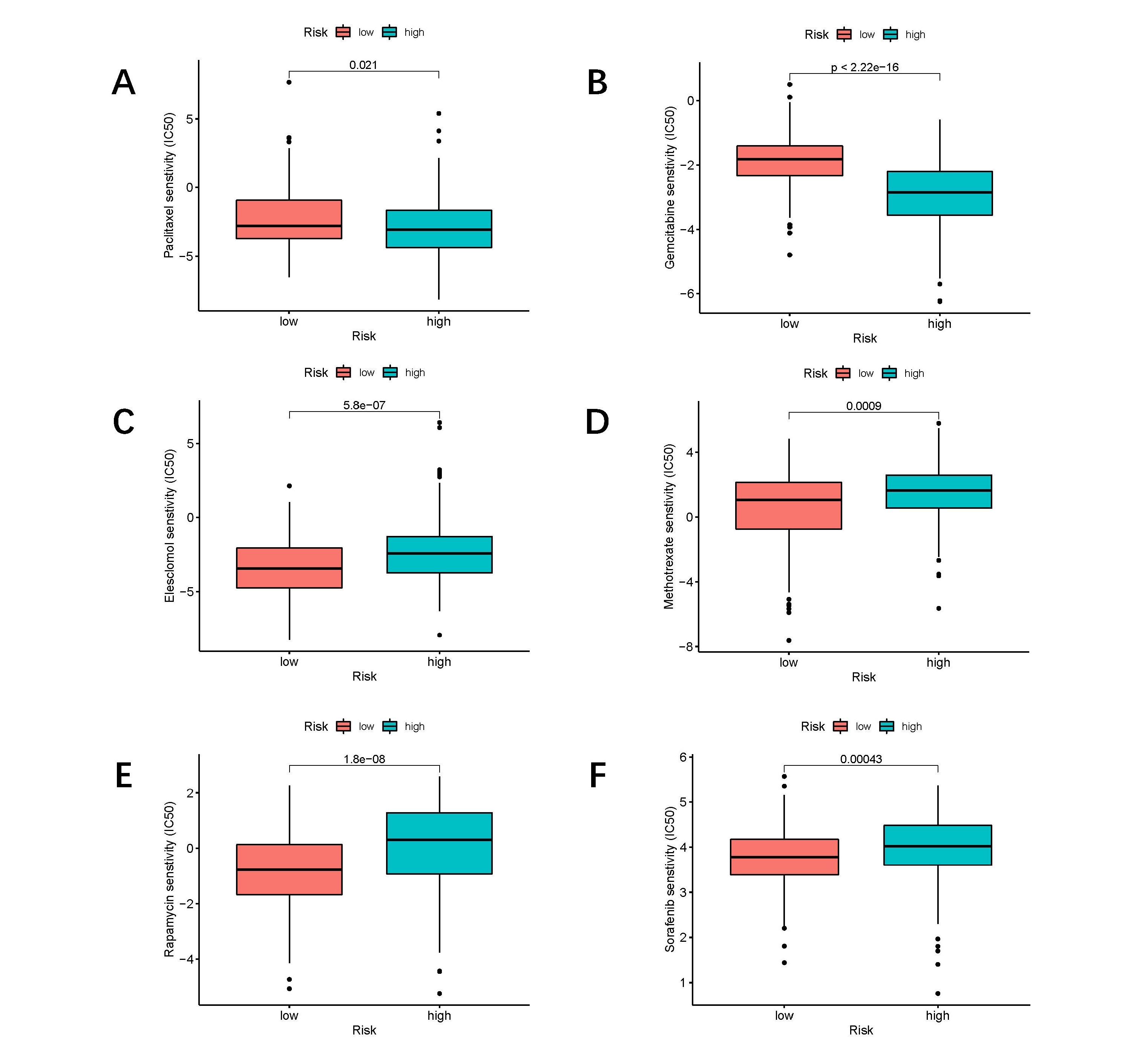

Supplement: Supplementary Figure 1 — Potential drug prediction. Boxplot showing the mean differences in estimated IC50 values of 6 representative drugs. (A)paclitaxel, (B)gemcitabine, (C)Elesclomol, (D)Methotrexate, (E)Rapamycin, and (F)Sorafenib. [file Image_1.jpeg]
